# Limited awareness of HIV Status hinders uptake of treatment among female sex workers and sexually exploited adolescents in Wau and Yambio, South Sudan

**DOI:** 10.1186/s12889-023-15593-z

**Published:** 2023-04-14

**Authors:** Alex Bolo, Patrick Ochira, Avi J. Hakim, Joel Katoro, Sudhir Bunga, Richard Lako, Victoria Anib, Golda Caesar Arkangelo, Brenda Nyokani Lobojo, Alfred G. Okiria

**Affiliations:** 1 Division of Global HIV and TB (DGHT), United States Centers for Disease Control and Prevention, Juba, South Sudan; 2 IntraHealth International, Juba, South Sudan; 3 United States Centers for Disease Control and Prevention, DGHT, Atlanta, GA USA; 4 South Sudan Ministry of Health, Juba, South Sudan

**Keywords:** RDS, Sex workers, Survey, South Sudan

## Abstract

**Background:**

Several factors determine uptake of HIV testing services (HTS) by female sex workers (FSW), including their knowledge of HIV and their awareness of services supporting people who are HIV-positive. HTS provided entry into the UNAIDS 90-90-90 cascade of care. We conducted a cross-sectional biobehavioural survey (BBS) to determine HIV prevalence and progress towards UNAIDS 90-90-90 cascade targets among this population in South Sudan.

**Methods:**

Respondent-driven sampling (RDS) was used to recruit women and sexually exploited girls aged 13–18 years who exchanged sex for goods or money in the past 6 months and resided in the town for at least 1 month. Consenting participants were interviewed and tested for HIV and, if positive, they were also tested for their viral load (VL). Data were weighted in RDS Analyst and analyzed with Stata 13.

**Results:**

A total of 1,284 participants were recruited. The overall HIV cascade coverages were 64.8% aware of their HIV-positive status; 91.0% of those aware of their positive status were on ART; and VL suppression among those on ART was 93.0%.

**Conclusion:**

Being unaware of their HIV-positive status limits, the uptake of HIV treatment among FSW in South Sudan. This underscores the importance of optimized case-finding approaches to increase HTS among FSW and sexually exploited minors.

## Introduction

In 2013, the Joint United Nations Programme on HIV/AIDS (UNAIDS) established the 90-90-90 targets to help end HIV by 2030. The expectation was that by 2020, 90.0% of people living with HIV would know their status; of those, 90.0% would be receiving antiretroviral therapy (ART); and of those, 90.0% would be virally suppressed [1]. Key populations and their sexual partners accounted for 60% of the 1.7 million (95.0% CI: 1.2–2.2 million) new global HIV infections in 2019 while 8.0% were specifically female sex workers (FSW) [2]. HIV prevalence in FSW [3] is about 2.2% (95% CI: 1.37–3.17%) globally and approximately 36.9% (95% CI: 36.2–37.5%) in sub-Saharan Africa [4]. Data regarding the HIV burden among FSW in South Sudan are limited [3, 5]. FSW in North Africa had a low mean HIV prevalence (1.4% [95% CI: 1.1–1.8%]) [6]. But HIV prevalence by country was second highest among FSW in South Sudan (17.3% [95% CI: 8.7–28.1%]) after Djibouti (17.9%). FSW in Juba, the capital of South Sudan had high HIV prevalence of 37.8% [7].

The FSW in Juba were diverse in nationality [7], including Ugandans (39.8%), South Sudanese (34.8%), Congolese (20.7%), and Kenyans (4.4%). The median age of FSW in Juba was 29 years (interquartile range [IQR], 25–34 years) [7]. The HIV prevalence in FSW and sexually exploited adolescents compared to female adolescents and women aged 15–49 years in the general population was 14.0 times higher in Juba and 13.5 times higher in Sub-Saharan Africa [8].

Several factors determine FSW access to HIV testing services (HTS), including knowledge about HIV, privacy and confidentiality, stigma and discrimination in the community and by healthcare providers, legal environment, education level, and poverty [2, 9–12]. A review of the literature of fifteen studies conducted in Sub-Saharan Africa regarding FSW access to HTS revealed how stigma related to sex work and HIV serves as major barrier to the uptake of HTS [9]. Legalizing sex work [13] and improving the acceptability of the HIV services offered by tailoring HTS services to adress FSW needs [14–16] such as having convenient service hours, short waiting times, and privacy may motivate FSW to seek HTS, which is a critical first step toward the attainment of the 90-90-90 targets [9]. Awareness of HTS services, its importance, and HIV risk perception influence FSW’s decision to undergo HIV testing [9, 14]. Empowering FSW with knowledge about HIV and explaining their high risk of HIV acquisition can motivate FSW to test for HIV [11]. However, only 12.5% (95% CI: 10.1–14.9%) of FSW in Juba had comprehensive knowledge of HIV [7] compared to 53.2% (95% CI: 49.6–56.5%) of FSW in Rwanda [17]. Although about three-quarters (78.8%) of FSW in Juba reported having tested for HIV, only 63.2% of 333 HIV-positive FSW knew their HIV status [7]. This level of HIV-positive status awareness is higher than the level reported in Rwanda (51.0%) [17] and Nairobi, Kenya (51.0%) [18].

We conducted cross-sectional biobehavioural survey (BBS) to determine HIV prevalence and characterize progress toward 90-90-90 targets among FSW in two South Sudan towns of Wau (2019), and Yambio (2019). We measured progress towards the 2020 Global 90-90-90 cascade given that the survey data was collected in 2019, outside the timeline to achieve the newly established 95-95-95 targets by 2025 [19]. No BBS in FSW had ever been conducted in Wau and Yambio, yet HIV service providers report sex workers being active. BBS in FSWs were already undertaken in Juba (2016) [7, 10] and Nimule (2017) [10] and published [7, 10]. The findings from all these surveys were used to plan HIV prevention, care, and treatment services for FSW. We present here the recent BBS findings from Wau and Yambio.

## Methods

### Study population and design

Respondent-driven sampling (RDS) was used to recruit participants for the BBS in two of the ten state capital towns in South Sudan of Wau (August–September 2019) and Yambio (October-November 2019). Eligibility criteria included females ages ≥ 13 years, who exchanged sex for goods or money in the previous 6 months, who resided in either Wau or Yambio for at least one month, and who had possession of a valid recruitment coupon. The existence of sexual exploitation of adolescents under 18 years in South Sudan [7, 20] justified their inclusion. Sexual exploitation refers to the practice of individuals with power who deceive children under 18 years into sexual activity in exchange of money or goods [21]. Therefore, participants included FSW ages 18-years and above and sexually exploited adolescents aged 13–17 years who have no ability to make informed decision and are deceived into having sex for money or goods. The three-source capture-recapture method was used to estimate FSW population size. The three-source data is from keychains and bangles distributed at different times and the BBS survey interview. Comprehensive knowledge about HIV was based on correct responses to five UNAIDS facts and myths about HIV.

### Recruitment and data collection

To estimate FSW population size, two different peer volunteer groups distributed two types of unique objects (keychains and bangles) to FSW and sexually exploited adolescents. The distribution started with keychains, followed a week later by bangles. In Wau, 958 FSW and sexually exploited adolescents received key chains and 953 received bangles. While in Yambio, 997 FSW and sexually exploited adolescents got keychains, and 999 received bangles.

One week after the distribution of bangles, oral interviews and biological testing began for eligible participants with consent. Propagation of recruitment was through initial FSW referred to as *seeds*, eight in Wau and seven in Yambio. The seeds were not from the peers who distributed the unique objects for population size estimation. Instead, the seeds were a different set of peers selected based on their nationality, age, influence, and residence duration in the towns. Each of the seeds and subsequent peers who completed the survey received three coupons to recruit peers who had not participated in the BBS.

Female study staff interviewed consenting participants using a structured questionnaire in Open Data Kit programmed tablets. The questionnaires were translated into Juba Arabic, Kiswahili, Zande, and Amharic and focused on participants’ demographics, knowledge of HIV, awareness of their HIV status, being on ART treatment, information about their sexual partners, and their use of condoms. Possessing comprehensive knowledge of HIV was based on the correct response to all five standard UNAIDS questions [22], which assessed participants’ knowledge of HIV being prevented by using a condom during sex; limiting sex to one faithful and uninfected partner; knowing a person who looks healthy can transmit HIV; mosquito bites cannot transmit HIV; and HIV cannot be transmitted by sharing food with persons infected with HIV.

Participants who consented to HIV testing were tested at the study site using the national rapid testing algorithm (Determine HIV-1/2 as screening test and Uni-Gold to confirm HIV-positive results). Additionally, dried blood spot samples were collected for Viral Load (VL) testing at the National Public Health Laboratory using Abbott m2000sp. Participants received a maximum of $14: transport refund ($3), time compensation ($5), and a $2 incentive for recruiting each peer.

### Data analysis

Survey data and serologic testing data were entered into password-protected tablets, and the files were encrypted. Data were stored on a network shared drive only accessed by authorized project staff. Data were cleaned using Excel and automatically weighted in RDS Analyst (RDS-A) where results were generated for proportions and confidence intervals. For those testing HIV-positive during the survey, the HIV cascade for self-reported HIV-positive status awareness, being on ART, and VL suppression (VL < 1,000 copies/ml) was developed using Stata and adjusted to the estimated PLHIV population in the study towns that was determined during the survey. Using data exported from RDS-A to Stata, bivariate logistics regression was applied to selected varriables to determine their level of influence in knowing HIV positive status for the HIV positive participants. Odds ratios for the correlates of knowing HIV-positive status were generated. During the study, data were monitored for convergence of population size estimate using RDS-A. Convergence was reached when increasing the sample size did not result in any further change in population size estimate. Data for both towns were pooled together to calculate combined estimates of interest.

### Ethical consideration

The protocol was reviewed and approved by the Government of South Sudan’s Ministry of Health Research Ethics Committee. The project was reviewed based on CDC human research protection procedures and was determined to be research. CDC investigators did not interact with human subjects or access identifiable data or specimens for research purposes. Participants provided separate verbal informed consent, first for interviews and later for HIV testing. Participants can decline HIV testing even after completing the interview. To ensure confidentiality, we did not collect names or other personal identifying data. For participants under 18 years, parental permission waivers were granted by South Sudan’s Ministry of Health Ethical Review Board. Adolescents under age 18 were provided with referrals to pre-determined organizations offering counseling, protection, and other relevant services for victims of sexual exploitation and gender-based violence.

## Results

The period taken to reach convergence point which influenced stopping data collection was seven weeks in Wau and four weeks in Yambio. We recruited 679 participants through 16 waves from the initial eight seeds in Wau and 605 participants from seven initial seeds through 12 waves in Yambio. Participant median age was 24 years (IQR, 20–30 years) in Wau, higher than for Yambio at 21 years (IQR, 19–25 years). In both towns, participants’ median age at the time of their first sexual experience was 15 years (IQR, Wau 14–17 and Yambio 13–15). In both towns, most participants were under 25 years, with about one-third being adolescents 15–19 years in Yambio and close to a quarter in Wau (Table 1).

**Table 1:** Participant’s demography, sexual history, and use of HIV related services

	Wau town (N = 679)			Yambio town (N = 605)		
Variables	n	%	95% CI (%)	n	%	95% CI
**Participant’s age (years)**						
< 15	2	0.2	0.0-0.4	4	0.7	0.0-1.4
15–19	153	23.7	18.4–29.0	162	29.8	24.8–34.8
20–24	188	28.7	23.0-34.3	231	40.1	35.2–45.0
< 25	343	52.6	45.2–60.1	397	70.6	65.7–75.6
≥ 25	335	47.4	40.0-54.8	206	29.4	24.4–34.3
**Participant’s nationality**						
South Sudanese	615	92.6	88.7–96.7	586	97.2	95.8–98.5
Sudanese	14	2.7	1.0-4.5	0	0.0	0.0
Ugandans	32	2.4	0.6–4.2	0	0.0	0.0
Congolese	18	2.2	0.0-4.4	17	2.6	1.3–3.9
Others	0	0.0	0.0	2	0.2	0.0-0.5
**Marital status**						
Divorced	297	44.0	38.1–49.9	81	12.4	10.0-14.9
Single / never married	272	39.4	33.0-45.7	505	84.5	81.8–87.2
Widowed	89	12.8	9.3–16.3	19	3	1.6–4.5
**Participants sexual history and income**						
Paid for their 1st sexual encounter	334	50.6	44.9–56.4	391	64.3	60.0-68.6
Raped at 1st Sexual encounter	197	28.4	23.2–33.7	71	12.2	9.2–15.3
Sexwork as their main source of income	626	91.5	88.8–94.2	548	89.6	86.8–92.4
**Participants access to HIV services and knowledge of HIV**						
Talked about HIV with an HIV outreach worker	230	32.6	27.9–37.4	143	22.7	18.7–26.6
Knew where to access HTS services	441	63.7	57.8–69.5	512	85.1	80.7–89.5
Previously tested for HIV	456	66.0	60.4–71.7	524	85.6	82.5–88.7
Reported HIV-positive previous test result	24	3.7	1.8–5.4	70	11.8	8.6–15.0
Comprehensive knowledge of HIV	117	17.7	14.2–21.2	15	2.6	1.3-4.0
Main reasons for previous HIV test*						
To know HIV status	167	32.9	26.7–38.8	301	56.8	51.3–62.2
Was pregnant	195	44.8	38.6–51.3	110	21.9	17.1–26.6
Reasons of not testing for HIV previously*						
No time	36	23.8	15.1–32.0	8	14	6.4–27.9
Fear of HIV-positive results	23	16.4	8.4–24.4	23	49.2	26.7–72.3
Cost barrier	22	18.1	9.2–27.3	1	1.4	0.2–9.8
No perceived HIV risk	10	5.9	0.0-12.6	7	12.5	1.6–23.2
**Condom use and barriers to its use**						
Used condom during last sexual encounter with cash clients	299	40.8	34.1–47.4	312	50.7	45.8–55.7
Not used condoms due to cost barrier	122	17.9	14.5–21.2	147	23.5	19.9–27.0
Do not know where to get condoms	170	27.0	20.9–33.0	19	3.5	1.8–5.1
Condoms not convenient	64	11.5	8.4–14.7	25	4.3	2.4–6.3

‘n’ unweighted absolute participant numbers, (%) weighted percentages, CI weighted 95% confidence interval. “N” is the total number of participants, except for the varriables marked with “*” which applies to a subset of N

The participants were predominately South Sudanese, (92.6%) in Wau and 97.2% in Yambio. Other nationalities were Sudanese, Ugandans, or Congolese (Table 1). The estimated population for FSWs including sexually exploited adolescents was 3,000 in Wau (95% CI: 2,203-6,079) ands 5,400 in Yambio (95% CI: 3,700-8,600).

Literacy, the ability to read and write, was only 22.7% in Wau and 37.8% in Yambio. Many (44.0%) participants were divorced in Wau compared with only 12.4% in Yambio. The median duration of participants residence in Wau was 13 years (IQR 6–22) and for Yambio 14 years (IQR 5–21). The main source of income for participants was sex work with more than half of participants paid at first sex, while some reported their first sexual encounter as rape (Table 1).

All 1,248 participants agreed to HIV testing in both towns. Of the 679 participants in Wau, 52 tested HIV-positive (23 knew they were HIV positive and 29 were new positives), resulting in a weighted HIV prevalence of 6.7%. In Yambio, 94 of 605 participants tested HIV-positive (68 knew they were HIV positive and 26 were new positives) giving a weighted HIV prevalence of 13.6%. About one-third (32.6%) of participants had ever talked about HIV with an HIV outreach worker in Wau, while in Yambio, only 22.7% had spoken with an outreach worker about HIV. In Wau, nearly two-thirds (63.7%) of participants knew where to access HTS, and two-thirds (66.0%) had previously tested for HIV. While in Yambio, most participants (85.1%) knew where to access HTS with 85.6% previously tested for HIV.

The main reasons for undergoing HIV testing in Wau were being pregnant (44.8%) and wanting to know their HIV status (32.9%). In Yambio, previous HIV testing was mainly for the purpose of knowing their HIV status (58.0%) followed by pregnancy 21.9%. In both towns, more than two-thirds of participants previously tested for HIV were tested at a health facility, 69.1% for Wau and 77.0% in Yambio. Barriers to HIV testing (Table 1) include lack of time, fear of an HIV-positive test result, associated testing cost and no perceived risk of being HIV infected.

Overall, 64.8% of the 146 HIV positive FSW in Wau and Yambio indicated self-reported knowledge of HIV-positive status with 91.0% of them on ART and 93.0% of those on ART having achieved viral suppression. Wau has lower awareness of participants’ HIV-positive status (35.4%) compared to Yambio (73.0%). In Yambio, the HIV cascade among all South Sudanese participants was much like that of all participants (Table 2). While knowledge of HIV-positive status and ART coverage are similar among South Sudanese and all participants in Wau, the VL suppression was lower among the South Sudanese compared to all the participants possibly due to low adherence to treatment among the South Sudanese enrolled on ART in Wau (Table 2).

**Table 2:** HIV Cascade for Participants in Wau and Yambio towns

	HIV Prevalence, n (%) 95% CI	1st 90: Awareness of HIV Positive status, n (%) 95% CI	2nd 90: Treatment Status for those HIV Positive n (%) 95% CI	3rd 90: VLS for those on ART and all HIV-positive survey participates, n (%) 95% CI	
		Self-reported awareness of HIV-positive status (D1)	Self- reported on ART (D2)	Suppressed VL for self-reported HIV-positives on ART (D3)	Suppressed VL for participants testing HIV-positive (D1)
Wau (all participants)	52 (6.7%) 4.1-9.4%	23 (35.4%) 15.9-51.9%	23 (100.0%)	19 (91.3%) 6.4 – 100.0%	37 (65.0%) 45.0-80.8%
Yambio (all participants)	94 (13.6%) 10.6-16.5%	68 (73.0%) 62.5 -83.4%	62 (89.8%) 81.0-98.5%	55 (93.2%) 88.5-97.9%	77 (86.2%) 77.6-91.8%
Wau and Yambio (all participants)	146 (11.2%) 9.3-13.4%	91 (64.8%) 55.0-73.6%	85 (91.0%) 80.1-96.2%	74 (93.0%) 85.9-96.6%	114 (81.6%) 73.2-87.7%
Wau (South Sudanese participants only)	32 (5.4%) 3.6-8.1%	14 (32.7%) 17.3-52.8%	14 (100.0%)	14 (100.0%)	22 (59.5%) 35.8-79.5%
Yambio (South Sudanese participants only)	91 (13.7%) 10.9-17.0%	65 (73.2%) 60.5-81.6%	59 (89.5%) 76.8-95.6%	52 (93.0%) 84.2-97.0%	74 (85.5%) 77.0-91.6%
Wau and Yambio (South Sudanese participants only)	123 (10.8%) 8.9-13.1%	79 (63.3%) 54.8-74.6%	73 (90.4%) 78.8-96.0%	66 (93.6%) 85.8-97.3%	96 (81.2%) 72.2-87.7%

“D” represents denominator, it varies by row, example for Wau and Yambio (all participants): D1: Participants who tested HIV−positive during the survey (146); D2 self−reported aware of HIV−positive status (91) and D3 self−reported HIV−positive participants on ART (91)

Analysis for correlates of knowing HIV-positive status (Table 3) indicates that in Yambio, young participants under 25 years were likely to know their HIV-positive status (AOR, 4.69 [95% CI: 1.02–21.54]. Ever talking about HIV with outreach worker does not increase likelihood of knowing HIV-positive status in both towns (AOR, 1.3 [95% CI: 0.38–4.50]. There is low likelihood of knowing HIV-positive status for clients who are not able to read and write in both towns, (AOR, 0.52 [95% CI: 0.19–1.40] though not statistically significant.

**Table 3:** Correlates of knowing HIV positive status for the HIV positive cases identified in the survey

	Wau (n = 52)						Yambio (n = 94)						Wau and Yambio combined (n = 146)					
	OR			AOR			OR			AOR			OR			AOR		
Variable	OR	95% (CI)	p-value	AOR	95% (CI)	p-value	OR	95% (CI)	p-value	AOR	95% (CI)	p-value	OR	95% (CI)	p-value	AOR	95% (CI)	p-value
South Sudanese	0.17	0.08–0.38	0.00	1.40	0.17–11.50	0.75	1.00			0.00		**0.31**	1.34	0.44–4.09	0.60	1.38	0.36–5.24	0.63
Unable to read and write	0.56	0.24–1.30	0.18	0.19	0.03–1.52	0.08	0.77	0.24–2.51	0.67	0.80	0.22–2.93	0.73	0.83	0.32–2.14	0.70	0.52	0.19–1.40	0.19
Age 13–24 years	0.32	0.15–0.65	0.00	0.10	0.01–1.52	0.04	2.47	0.76–8.01	0.13	4.69	1.02–21.54	0.03	1.69	0.72–3.96	0.23	1.29	0.53–3.16	0.58
Talked about HIV with outreach worker	1.15	0 0.59-2.27	0.68	0.93	0.10–8.53	0.95	2.23	0.56–8.83	0.254	1.67	0.30–9.39	0.55	0.97	0.40–2.34	0.94	1.30	0.38–4.50	0.68
Know where to go for HIV test	2.07	0.94–4.55	0.07			0.41	5.16	0.90-29.48	0.065	3.46	0.64–18.83	0.13	3.46	0.93–10.7	0.04	2.71	0.64–11.42	0.16
Has comprehensive HIV knowledge	0.96	0.42–2.18	0.92	0.21	0.02–2.41	0.17	1.00			0.00		0.76	0.62	0.15–2.61	0.52	0.16	0.02–1.07	0.03
Afraid to seek health care due to selling sex	0.80	0.38–1.70	0.56	0.33	0.01–10.58	0.51	0.31	0.07–1.33	0.12	0.00		0.01	0.41	0.14–1.21	0.11	0.09	0.01–0.71	0.00
Ever been pregnant	2.01	0.74–5.44	0.17	0.00		0.56	1.78	0.45–7.02	0.41	1.68	0.50–5.61	0.39	1.19	0.37–3.83	0.77	1.25	0.39–3.97	0.71

(1) Unadjusted odds ratio (OR) and Adjusted odds ratio (AOR) (2) For each of the variables, the OR was adjusted for the other variables in Table 3. (3) For OR 1:00 Stata returned zero value for CI, and AOR. There are also instances where Stata returned blank for AORs and some p-values. The OR or the AOR can be infinite when the denominator is zero and the resulting OR/AOR will be missing.

Comprehensive HIV knowledge was only 17.7% in Wau and just 2.6% in Yambio. Participants’ use of condoms during their last sexual encounter with cash clients was only 37.2% in Wau and 40.8% in Yambio. The most common barrier to condom use was not knowing where to obtain them, cost, and inconvenience of their use (Table 1).

## Discussion

Of the 146 HIV positive participants in Wau and Yambio only 64.8% were aware of their status. Most (91.0%) of those aware of their HIV-positive status reported being on ART with 93.0% VL suppression. Participants from Wau had lower HIV cascade coverages than Yambio, especially for self-reported awareness of HIV-positive status, which was 35.4% in Wau compared with 73.0% in Yambio (Table 2). The selef-reported awareness of HIV-positive status in Yambio is comparable to that of Juba (74.8%) and Nimule 79.5%) [23]. We did not specifically explore the reasons for such differences in coverages. Lower proportions of self-reported knowledge of HIV-positive status have also been found in FSWs in Juba, 36.8% [7], lower than in Nairobi (51%) [18], Zimbabwe (64%) [24]. Therefore, knowledge of HIV-positive status is a limiting factor for comprehensive HIV care and treatment for FSW and sexually exploited adolescents in South Sudan and could lead to increased new HIV infections.

While some participants in both towns have talked about HIV with outreach workers, and most know where they can be tested, there is a need to ensure all FSW and sexually exploited adolescents have access to HIV information and HTS is available to them. By doing so the likelihood of HIV-positive FSW and sexually exploited adolescents knowing their HIV status and treatment enrollments can be increased. Our study provided an opportunity to diagnose and link to ART 55 new HIV positive cases, 29 in Wau and 26 in Yambio. South Sudan requires coordinated efforts of partners to expand targeted HIV messaging and acceptable HIV testing strategies in major towns to reach FSWs and sexually exploited adolescents. It is also important to understand current HIV service strategies for FSWs and sexually exploited adolescents in Yambio which could be adopted in major towns in South Sudan.

The low literacy [25] in both towns may be contributing to the low awareness of HIV-positive status, especially in Wau town. Focus group discussions among FSW in Wau may help uncover additional explanations for the very low awareness of HIV-positive status. The low HIV awareness could be addressed by providing appropriate HIV messages through peers. The low level of education among FSW and sexually exploited adolescents in South Sudan [7], with few discussing about HIV with community health workers results in very low comprehensive knowledge of HIV and less use of HTS. Innovative approaches such as peer-to-peer network referral [15, 26–28] and tailored easy to understand education materials could help improve HIV knowledge and increase access to HTS for this population. Though at an extra cost, dedicated HIV service delivery points outside of health facilities tailored for FSW in major towns in South Sudan can enhance access to HTS [15, 26–29].

FSW and sexually exploited adolescents are predominantly South Sudanese in Wau (92.6%) and Yambio (97.2%) compared to the capital Juba (34.8%) which could be having a bearing on the HIV prevaence in FSWs. The 6.7% HIV prevalence among FSW and sexually exploited adolescents in Wau is far lower than the 13.6% in Yambio and 37.8% prevalence in the capital Juba, [11]. The HIV prevalence in the same population in the capital cities of neighboring countries - Nairobi, Kenya [18] (29.5%) and Kampala, Uganda [30] (33.0%) - are slightly below that of Juba. Nevertheless, it is possible the HIV epidemic in this population in South Sudan is expanding, and the delivery of surveillance and tailored service could help improve outcomes. We postulate that Yambio located in high disease burden Western Eauatoria state and also neighboring Democratic Republic of Congo with high general population HIV prevalence estimated at 11.0% for Kinshasa [31] combined with prevailing cultural sexual practices may contribute to the higher HIV prevalence in Yambio compared to Wau which is located closer to the border with Sudan which has lower HIV prevalence [6].

We encountered 6 children under 15 years in the study who are considered sexually exploited and were linked to providers of sexual and gender-based violence service, though we did not follow the referral outcome. Children are highly vulnerable in post-conflict settings including South Sudan that suffered protracted armed conflict that disrupted development resulting in low social status and low literacy making these children vulnerable to sexual exploitation [21]. In South Sudan, having sex with a child under 15 years is a defilement offence punishable by law. However, the challenge is in getting the perpetrators. This finding, though with limited data highlights the existence of sexual exploitation of children in Wau and Yambio. Given the limited data, high level of stigmatization, discrimination, restrictive laws, and harassment of FSWs by government authorities in many countries [2, 32], we are unable to provide any specific recommendation to address the Sexual exploitation of children in these towns.

Our findings are subject to limitations. For example, self-reported previous HIV-negative test results may have recently turned positive. Yet, we did not conduct recency testing for recent HIV infections unknown to participants. In addition, the high VL suppression among all HIV-positive participants (87.7%) may imply some HIV-positive participants were on ART and reported they were HIV-negative. Given the small number of HIV positive cases (146), we did not do multivariate logistic regression to determination correlates of knowing HIV positive status but only did bivariate logistic regression limited to few variables and not the entire study varriables. With smaller HIV-positive cases per town Wau (52) and Yambio (94) we opted to separately analyse the corelates of knowing HIV status for each of these towns because these towns are located in different parts of the country with Wau to the North close to be border with Sudan while Yambio is to the South East close to Democratic Republic of Congo. Also, the social characteristics of the people living in these towns are different which could have resulted in the difference in HIV prevalence of only 6.7% in Wau and 13.0% Yambio. We did not separately analyse the VL suppression among the newly identified HIV positive participants (52) which if combined with recency testing could provide a clue on whether some of the participants were known HIV positives already on ART. It is important to include recency testing in BBS for key populations inorder to identify hotspots of new HIV infections and implement HIV prevention measures.

## Conclusion

Unlike in the capital city Juba, FSW and sexually exploited adolescents in Wau and Yambio were primarily South Sudanese with limited knowledge of HIV-positive status hindering access to HIV care and treatment. Therefore, to control the HIV epidemic in the country, the findings of this study indicate it may be of value to direct future efforts towards increasing HIV knowledge and access to acceptable HTS for FSW and sexually exploited adolescents in major towns in South Sudan.

## Data Availability

Study data are available on request by writing to Alex Bolo (abolouk45@gmail.com).
